# Identification of new biomarker candidates for glucocorticoid induced insulin resistance using literature mining

**DOI:** 10.1186/1756-0381-6-2

**Published:** 2013-02-04

**Authors:** Wilco WM Fleuren, Erik JM Toonen, Stefan Verhoeven, Raoul Frijters, Tim Hulsen, Ton Rullmann, René van Schaik, Jacob de Vlieg, Wynand Alkema

**Affiliations:** 1 Computational Drug Discovery (CDD), CMBI, NCMLS, Radboud University Nijmegen Medical Centre, P.O. Box 9101, 6500 HB, Nijmegen, The Netherlands; 2 Netherlands Bioinformatics Centre (NBIC), P.O. Box 9101, 6500 HB, Nijmegen, The Netherlands; 3 Department of Medicine, Radboud University Nijmegen Medical Centre, Nijmegen, The Netherlands; 4 Netherlands eScience Center, Amsterdam, The Netherlands; 5 TNO, Zeist, The Netherlands; 6 Present address: Rijk Zwaan Nederland BV, Fijnaart, The Netherlands; 7 Present address: Philips Research Europe, Eindhoven, The Netherlands; 8 Present address: NIZO Food Research BV, Ede, The Netherlands

**Keywords:** Literature mining, Insulin resistance, Glucocorticoids, Gene networks

## Abstract

**Background:**

Glucocorticoids are potent anti-inflammatory agents used for the treatment of diseases such as rheumatoid arthritis, asthma, inflammatory bowel disease and psoriasis. Unfortunately, usage is limited because of metabolic side-effects, e.g. insulin resistance, glucose intolerance and diabetes. To gain more insight into the mechanisms behind glucocorticoid induced insulin resistance, it is important to understand which genes play a role in the development of insulin resistance and which genes are affected by glucocorticoids.

Medline abstracts contain many studies about insulin resistance and the molecular effects of glucocorticoids and thus are a good resource to study these effects.

**Results:**

We developed CoPubGene a method to automatically identify gene-disease associations in Medline abstracts. We used this method to create a literature network of genes related to insulin resistance and to evaluate the importance of the genes in this network for glucocorticoid induced metabolic side effects and anti-inflammatory processes.

With this approach we found several genes that already are considered markers of GC induced IR, such as *phosphoenolpyruvate carboxykinase* (*PCK*) and *glucose*-*6*-*phosphatase*, *catalytic subunit* (*G6PC*). In addition, we found genes involved in steroid synthesis that have not yet been recognized as mediators of GC induced IR.

**Conclusions:**

With this approach we are able to construct a robust informative literature network of insulin resistance related genes that gave new insights to better understand the mechanisms behind GC induced IR. The method has been set up in a generic way so it can be applied to a wide variety of disease networks.

## Background

Glucocorticoids (GCs) are often prescribed for the treatment of inflammatory diseases such as rheumatoid arthritis, asthma, inflammatory bowel disease and psoriasis [1-3]. Despite their excellent efficacy, usage is limited because of side-effects such as insulin resistance, glucose intolerance, diabetes, central adiposity, dyslipidemia, skeletal muscle wasting and osteoporosis [4-8].

GCs bind to the glucocorticoid receptor (GR), which then dimerizes and translocates to the nucleus where it influences gene transcription. Positive regulation of genes (transactivation) is mainly mediated by direct binding of the GR-GC complex to glucocorticoid response elements located in the regulatory region of a target gene. The GR-GC complex may also bind to negative glucocorticoid response elements, which leads to a negative regulation of genes (transrepression). It is believed that transrepression, in which proinflammatory genes are downregulated, is mainly responsible for the efficacy of GCs as anti-inflammatory drugs [5,7], while transactivation might be responsible for the GC-induced adverse effects [9].

An important side effect is the development of insulin resistance (IR), because it is the onset of many metabolic diseases and conditions such as obesity, diabetes mellitus and hypertension. IR is a physiological condition in which a given concentration of insulin produces a less-than-expected biological effect. These biological effects are different depending on the tissue in which they occur. For instance, under IR conditions, fat and muscle cells fail to adequately respond to circulating insulin, which results in reduced glucose uptake, and subsequently higher glucose levels in blood [10,11]. In liver cells the IR- effects can be seen in reduced glycogen synthesis and storage, and a failure to suppress glucose production and release into the blood.

One way by which GCs induce IR is by inhibition of the recruitment of *GLUT4* glucose transporter, which results in reduced insulin-stimulated glucose transport in skeletal muscle [12]. However, not all mechanisms involved in GC-induced side effects are not completely understood. To gain more insight into mechanisms behind GC induced IR, it is important to understand which genes play a role in the development of insulin resistance and which genes are affected by GCs.

It has been widely recognized that a system approach in which networks of genes in their functional context are studied, contributes to a better understanding of the mechanisms and pathways related to the disease and the drug effects [13-17]. To study a gene network related to a disease such as IR, a list of disease related genes as well as a notion of the interactions between these genes is needed.

Literature databases such as Medline contain many studies about IR and the molecular effects of synthetic glucocorticoids and thus are a good resource that can be used to create and study disease related gene networks.

The retrieval of relevant gene-disease associations out of the millions of abstracts in Medline is very labor intensive and thus a text mining system is needed to this in an automated fashion.

In previous work we reported about CoPub [18-20], a publicly available text mining system, which has successfully been used for the analysis of microarray data and in toxicogenomics studies [21-26]. CoPub calculates keyword co-occurrences in titles and abstracts from the entire Medline database, using thesauri for genes, diseases, drugs and pathways. We used this technology to develop CoPubGene, a rapid gene – disease network building tool. To evaluate the importance of genes in these networks we implemented a method to score the importance of genes in biological processes of interest by incorporating their functional neighborhood.

We used CoPubGene to create a network of genes related to insulin resistance and to evaluate the importance of the genes in this network for glucocorticoid induced metabolic side effects and anti-inflammatory processes.

By using this method, we identified several genes that already are considered markers of GC induced IR, such as *phosphoenolpyruvate carboxykinase* (*PCK*) and *glucose6phosphatase*, *catalytic subunit* (*G6PC*) [27,28]. Even more importantly, we were able to identify genes involved in steroid synthesis that have not yet been recognized as mediators of GC induced IR.

## Methods

### CoPubGene

We constructed CoPubGene as a SOAP based web service (Table 1). This CoPub Web Service WSDL is created in Eclipse using the so-called Document Literal Wrapped style. The web service provider code is written in Perl using the SOAP::WSDL module and is available via the CoPub portal http://www.copub.org.

**Table 1:** List of available operations of the CoPub Web Service

**Name**	**Operation name**	**Input**	**Output**	**Description**
Get genes	*Get*_*genes*	Gene name, gene identifier	Biological identifier(s), with gene specific information	Each gene in CoPub belongs to an internal identifier (biological identifier). *Get*_*genes* converts the input gene to such a Biological identifier. This biological identifier serves as an input for subsequent operations.
Get Keywords	*Get*_*keywords*	Keyword	Biological identifier(s), with keyword specific information	Retrieves for a set of keywords, the Biological identifiers to which these keywords belong in CoPub. These biological identifiers serve as an input for subsequent operations.
Get references	*Get*_*references*	Biological identifier(s)	Literature references	Given a Biological identifier, retrieves all abstracts in which the term occurs.
Get literature neighbours	*Get*_*literature*_*neighbours*	Biological identifier(s)	Literature neighbors	Given a Biological identifier, retrieves a list of keywords which are mentioned in the literature together with the input term.
Get enriched keywords	*Get*_*enriched*_*keywords*	List of gene identifiers	List of enriched keywords	For a list of genes, this operation calculates a keyword overrepresentation.
Get literature network	*Get*_*literature*_*network*	Biological identifier(s)	SVG / Cytoscape network	For a set of genes, the operation creates a network of genes.
Get categories	*Get*_*Categories*	-	List of categories	Returns a list of categories of terms in CoPub
Get chips	*Get*_*chips*	-	List of microarrays	Returns a list of available Affymetrix chip names in CoPub.
Version	*Version*	-	Version of code and literature	Returns the version of the code and literature.
Selftest	*Selftest*	-	Diagnostic information	-

Biological identifiers are used by CoPub to identify biological concepts in the system. Each biological concept has a unique identifier.

### Retrieval of Gene-Disease associations

To create disease related gene networks, we used CoPubGene to retrieve gene-disease and gene-gene associations from Medline abstracts. Disease terms which had significant gene associations based on the R-scaled score (rs > 35) and literature count (lc > 5) in Medline abstracts, were extracted from the CoPub thesaurus.

### Disease clustering

Disease clustering was done in R (http://www.r-project.org) using the pvclust R package with “complete” setting for hierarchical clustering, based on correlation distance of R-scaled scores between genes and diseases, with 100 bootstrap replications. The hierarchical cluster was visualized using Denroscope [29]. Additional gene set enrichment analysis against the GENETIC_ASSOCIATION_DB_DISEASE was done with the annotation server DAVID [30,31].

### Creation of IR gene network

CoPubGene was used to create a set of genes related to IR, by searching for associations between genes and IR in Medline abstracts using default values (rs > 30 and lc > 5). Subsequently the IR-gene network was created by connecting genes that had significant co-occurrences with each other.

### Keyword enrichment analysis of IR related genes

Keyword enrichment analysis on the list of IR related genes was done against disease and drug terms from the CoPub database. Threshold values were chosen using default values.

### Analysis of the IR gene network and calculation of neighbor score for genes

The IR gene network was analyzed by mapping specific occurrences of the IR related genes with ‘inflammation’ and ‘dexamethasone’ in Medline abstracts onto the network. For the evaluation of the involvement of a gene, calculation of the literature score for a given gene and a given disease term, also the effects of dexamethasone and inflammation on the connecting genes are included. The literature score for *gene g* with *term d* is calculated in the following way:

(1) $$\mathrm{Literature}\_\mathrm{score}\_g=\frac{g1+\mathrm{Ns}}{2},\;\mathrm{Ns}=\frac{\left(\mathrm{rg}2*g2\right)+\left(\mathrm{rg}3*g3\right)+.\left(\mathrm{rgn}*\mathrm{gn}\right)}{\mathrm{rg}2+\mathrm{rg}3+.\mathrm{rgn}}$$

In which *g1* is the R-scaled score of *gene g* with *term d*, and Ns is the literature score of its neighboring genes with *term d*. This latter score *Ns* is calculated using the R-scaled score of each neighboring gene of *gene g* with *term d* (g2, g3,.,gn) relative to its relation (R-scaled score) with *gene g* (rg2, rg3,.,rgn).

## Results

We developed CoPubGene by creating a number of web service operations that can be used to construct networks of genes based on their co-occurrences in Medline abstracts. These web service operations can be combined to answer a variety of biological questions (Table 1). For example, the question “to what biological processes is this gene related?” can be answered by running the “get genes” and “get literature neighbours” functions. Using subsequently the “get references” function will return all the relevant pubmed entries in which the gene and keywords co-occur. By applying the “get keywords” and “get literature neighbours” functions one can retrieve all disease terms that are linked to a given drug term in the Medline abstract, or vice versa, retrieve all drug terms that are linked to a given disease term in abstracts. The networks that are created can be written to Cytoscape for downstream applications and visualizations. Also more advanced questions such as the construction of disease related gene networks, and subsequent calculation of keyword enrichment in this network can be addressed in an automatic way. In Table 1 the available web service operations are shown.

### Retrieval of gene-disease associations

Our aim was to get insight into the pathways and genes that are involved in insulin resistance, and the effect of glucocorticoids on this network. As a first step we created a list of genes associated with insulin resistance using CoPubGene. This yielded a list of 384 genes each of them connected to IR with an R scaled score (in Additional file 1: Table S2A the top scoring genes with IR are shown, the full list is available in Additional file 2: Table S2). To evaluate the quality of this list and to investigate whether this gene list is unique for IR or whether this list contains a large number of genes that are associated with multiple diseases we constructed a gene association list for all diseases in the disease thesaurus of CoPub, using similar parameter settings as used for construction of the IR gene list. This yielded a list of disease profiles with for each disease, a number of genes connected to that disease with an R scaled score. (Additional file 1: Table S2 shows the results for a few selected diseases, the full table is available in Additional file 2: Table S3).

**Figure 1:**
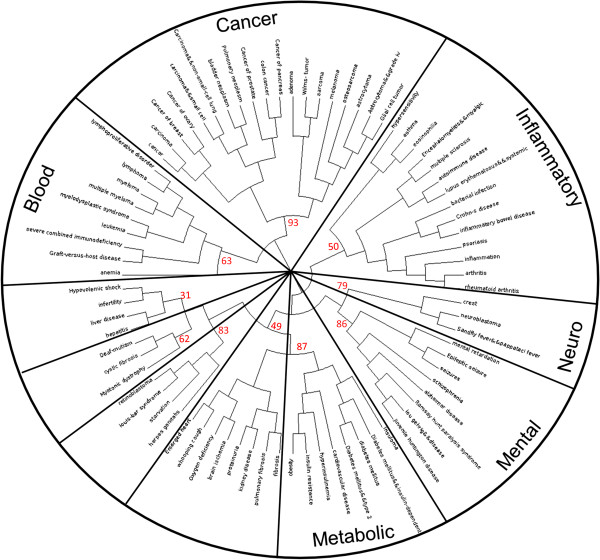
**Hierarchical cluster of disease terms from the CoPub database.** The top 80 disease terms with the most gene associations are shown. Disease terms are clustered together based on having the same gene associations. Red numbers at the nodes represent approximately unbiased bootstrap values (%).

These disease profiles were clustered using hierarchical clustering with multiscale bootstrap resampling, grouping together disease terms which have a similar profile, i.e. co-occur with the same genes (Figure 1; See Additional file 3: Figure S2 for the cluster with all bootstrap values). It appeared that a number of clusters of similar disease terms i.e. disease terms for which it is known that they have similar symptoms or have a similar mode of action, could be identified. For instance cancer related terms, such as ‘cancer of breast’, ‘cancer of prostate’ and ‘colon cancer’ are clustered together and inflammatory related disease terms such as ‘psoriasis’, ‘inflammatory bowel disease’ and ‘asthma’ are clustered together. These clusters also have high unbiased (AU) bootstrap values, indicating strong evidence for these clusters. To further confirm that the found gene-disease associations by CoPub are indeed biologically relevant, for each sub-cluster in Figure 1, we collected the union of all genes for that sub-cluster, and used these genes to perform a functional annotation analysis against the genetic association disease database using DAVID. The results of this analysis indicated that indeed similar disease terms to CoPub were found by DAVID (for the results of this analysis see Additional file 4). These analyses showed that with CoPubGene we are able to construct a relevant list of specific IR related genes that can be used for further analysis and that CoPubGene can be used to create a variety of disease related genes lists.

### Network of insulin resistance related genes

**Figure 2:**
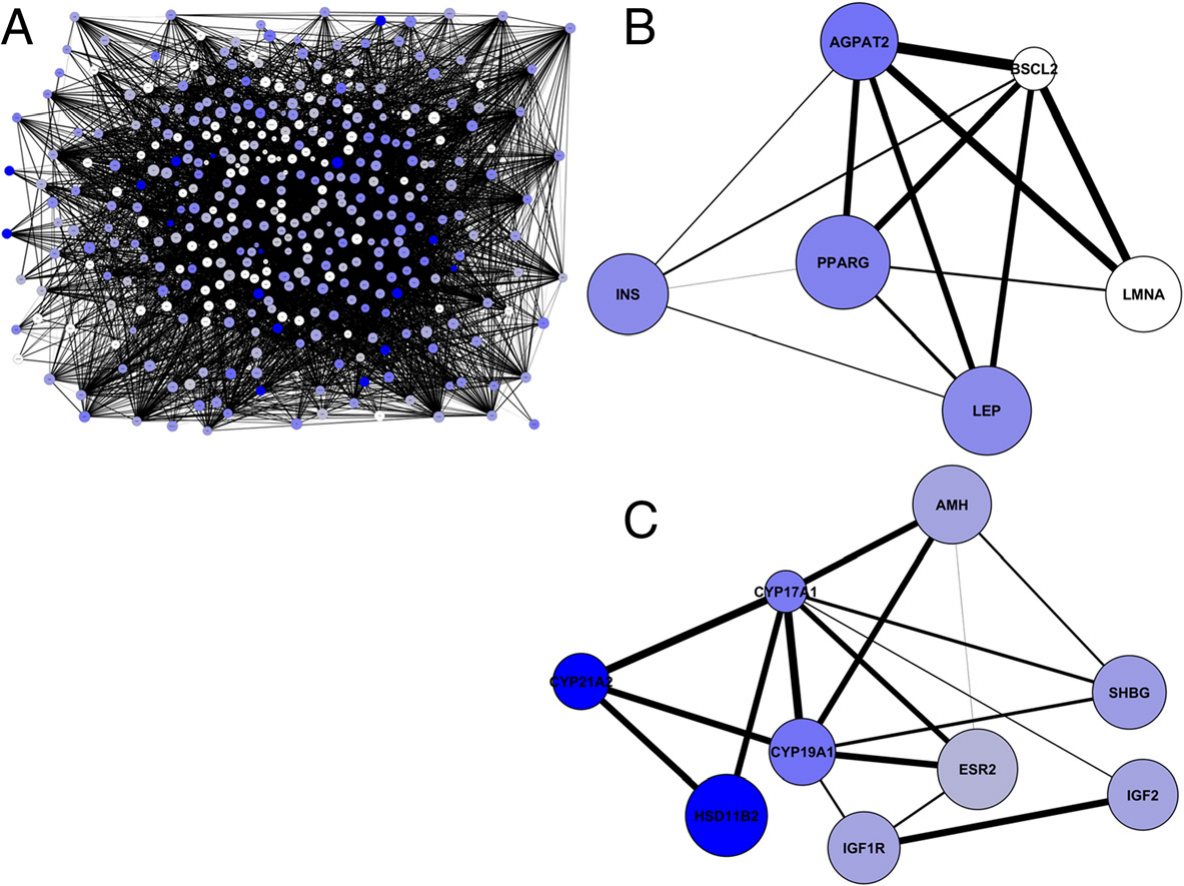
**Literature network of insulin resistance related genes (A).** Genes, represented by nodes are linked, based on co-occurrences in Medline abstracts. The thickness of the edge indicates the strength of the link between two genes (R-scaled score). Genes in blue have a co-occurrence with dexamethasone in Medline abstracts (R-scaled score). The strength of the link with dexamethasone is given by the color shading, ranging from no link (white) to a strong link (dark blue). The strength of the link with inflammation (R-scaled score) is given by the size of the node of the gene, ranging from no link (normal size of the node) to a strong link with inflammation (large size of the node). Sub-network for gene *PPARG* (**B**). Sub-network of Cytochrome P450s (**C**).

To create the IR gene network, we used the 384 genes from the IR gene list and connected the genes based on their co-occurrences with each other in Medline abstracts. The resulting network is shown in Figure 2A. We found that 381 genes of the IR gene list were connected to at least one other gene. We identified a number of hubs such as *peroxisome proliferatoractivated receptor gamma* (*PPARG*), *insulin receptor substrate 1* (*IRS1*), *vakt murine thymoma viral oncogene homolog 1* (*AKT1*), *insulin receptor* (*INSR*), *solute carrier family 2* (*facilitated glucose transporter*), *member 4* (*SLC2A4*) and *insulin* (*INS*) which were connected to more than 100 other genes. The resulting network is a scale free network, as indicated by the distribution of connectivity that follows a power law distribution which is indicative for a scale free network (Additional file 5: Figure S1) [32]. Although the above network has the characteristics of a biological network, and contains the expected genes as central hubs, without additional annotations this network representation is still largely uninformative and contains too little substructure to draw biological conclusions.

### Annotation of the network with drugs and diseases terms

As a first step towards annotating the network and identification of sub networks with a shared biological function, we investigated which drugs and diseases in the literature are specifically linked to this network using a keyword enrichment analysis on the list of IR related genes (For details about the enrichment method see Table 1). This enrichment yielded a number of drugs that are known drugs for the treatment of diabetes such as ‘rosiglitazone’, ‘metformin’ , ‘pioglitazone’, and also ‘glucagon’ and ‘insulin’ which are frequently used for the treatment of hypoglycemia and hypoinsulinemia (Table 2A. For the full list see Additional file 2: Table S4A). Notably, among these top scoring drugs we found dexamethasone, a well known synthetic glucocorticoid. High scoring genes with dexamethasone are for instance *CEBPA*, *SERPINA6*, *PCK2* and *GPD1* (for a full list of genes per enriched drug term, see Additional file 2: Table S4A.2), which also have been mentioned in the development of several metabolic diseases [33-37].

**Table 2:** **Over**-**represented drug and disease terms** (**P**-**value** < **0**.**05**)

**A**	
** Term**	**Number of genes**
insulin	358
dexamethasone	195
nitric oxide	193
estrogen	169
adenosine	151
estradiol	145
rosiglitazone	125
actinomycin	124
actinomycin d	121
glucagon	120
thrombin	108
progesterone	97
trypsin	86
nicotinamide	85
metformin	84
pioglitazone	82
**B**	
** Term**	**Number of genes**
insulin resistance	381
obesity	263
inflammation	219
diabetes mellitus	190
cardiovascular disease	181
Diabetes mellitus,type 2	173
Oxygen deficiency	164
fibrosis	138
hyperinsulinemia	137
Cancer of breast	131
Adiposity	130
cancer	128
starvation	120

The top scoring drug terms in the IR network from the CoPub database (**A**). Top scoring disease terms from the CoPub database in the IR network (**B**).

There are several top scoring over-represented terms that are related to metabolic diseases, e.g. ‘diabetes mellitus’, ‘obesity’, ‘diabetes mellitus, type 2’ and ‘hyperinsulinemia’ (Table 2B). The fact that these terms are high scoring is expected since we constructed the gene network based on the keyword insulin resistance. However we also found diseases that share a common origin with insulin resistance such as cardiovascular disease (Table 2B). The most interesting high scoring term for our particular research question was the non-metabolic term ‘inflammation’, which was represented in the network by genes such as *IL6*, *IL18*, *IL1RA*, *SOCS1*, *SOCS3*, *CCL2* and *CCR2*. Several of these genes have been mentioned in studies to be involved in the development of metabolic diseases. For instance, elevated levels of *IL6* in subjects with obesity and diabetes showed an association between insulin resistance and IL6 [38]. Studies in mice showed that *CCR2* deficiency or antagonism of this receptor resulted in attenuation of systemic insulin resistance and development of obesity, hence suggesting a modulating role of *CCR2* in this [39,40].

These results show that even with an unbiased data driven construction of a gene network, the relation between IR, dexamethasone and inflammation is discovered based on the genes that play a role in these effects. We subsequently highlighted the genes in the IR network that are related to inflammation and dexamethasone (Figure 2).

### Genes linked to inflammation and glucocorticoids in the context of insulin resistance

**Figure 3:**
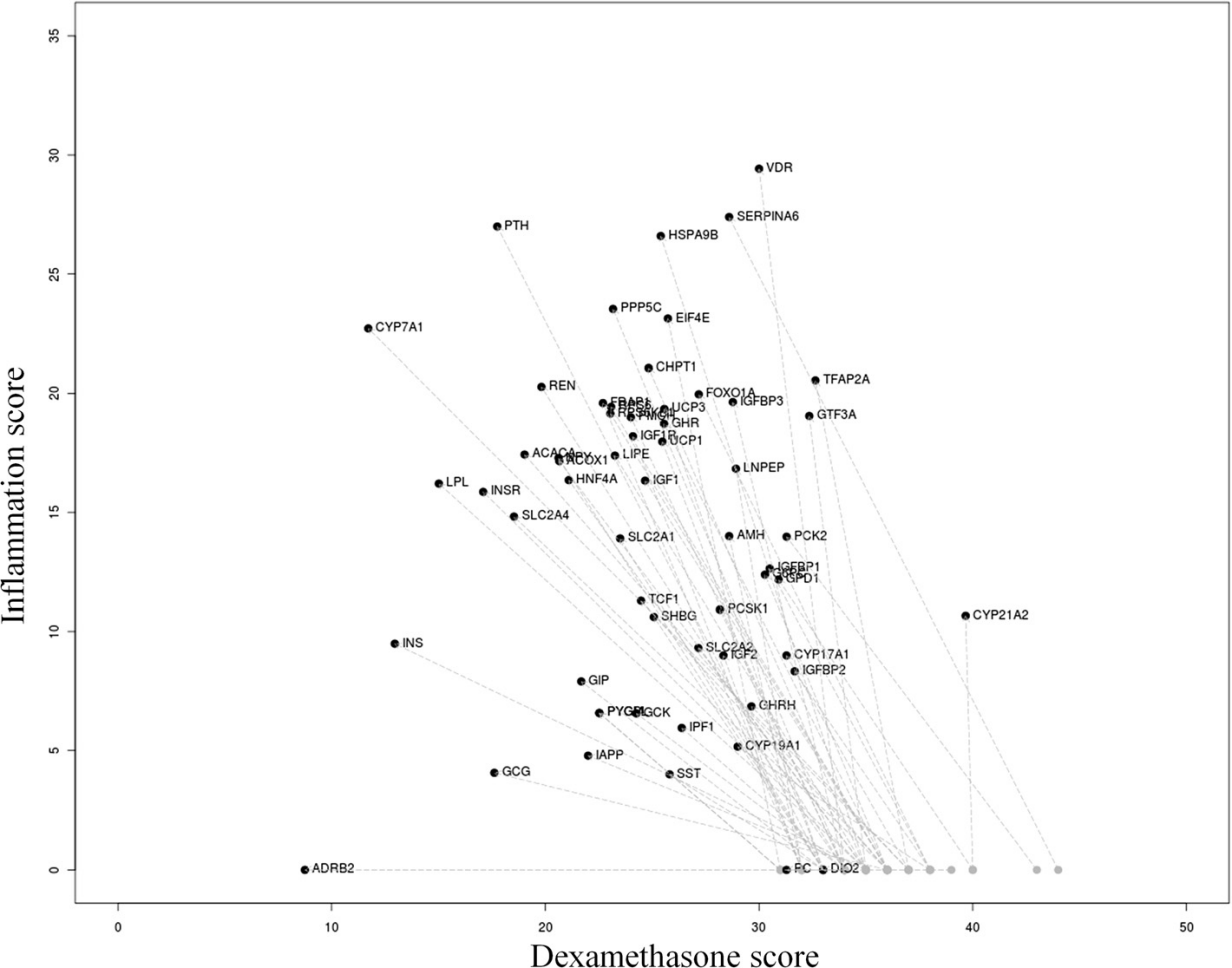
**Influence of dexamethasone and inflammation on IR genes that have a high score with dexamethasone (>25)and a low score with inflammation (<25). **The direct score of these genes with dexamethasone and inflammation are shown in grey. The literature neighbor score for these genes, by also including the relations of dexamethasone and inflammation with genes to which the gene is connected in the network, are shown in black. The grey arrows indicate the migration of the gene from a direct score to a literature neighbor score.

From a drug development perspective it is interesting to separate the desired effect of GCs on inflammatory processes from the undesired effect on metabolic processes. To rank each gene with respect to the relation with GC and inflammation, we calculated for each gene a literature score with dexamethasone and inflammation. Subsequently we focused on genes that score low on inflammation and high on dexamethasone (Figure 3). These genes are thought to be more exclusively related to GC induced IR. For these genes we calculated a literature neighbor score as well, by also including the relations of dexamethasone and inflammation with genes to which the gene is connected in the network. In Figure 3 it is shown that many genes which are not directly connected to inflammation (grey dots) are definitely influenced by inflammation via their connecting genes (black dots).

The majority of the genes in Figure 3 are directly involved in important metabolic processes such as gluconeogenesis (*PCK2*, *G6PC*, *PC* and *GCG*), glycolysis (*GCK*, *GCG*), glucose uptake, lipid metabolism (*ACACA*, *CHPT1*, *GPD1*) and carbohydrate metabolism (*GPD1*). Other ones are directly involved in insulin signaling (*GIP*, *IGF2*, *IPF1*, *IAPP*).

### Sex steroid physiology in relation to insulin resistance

**Figure 4:**
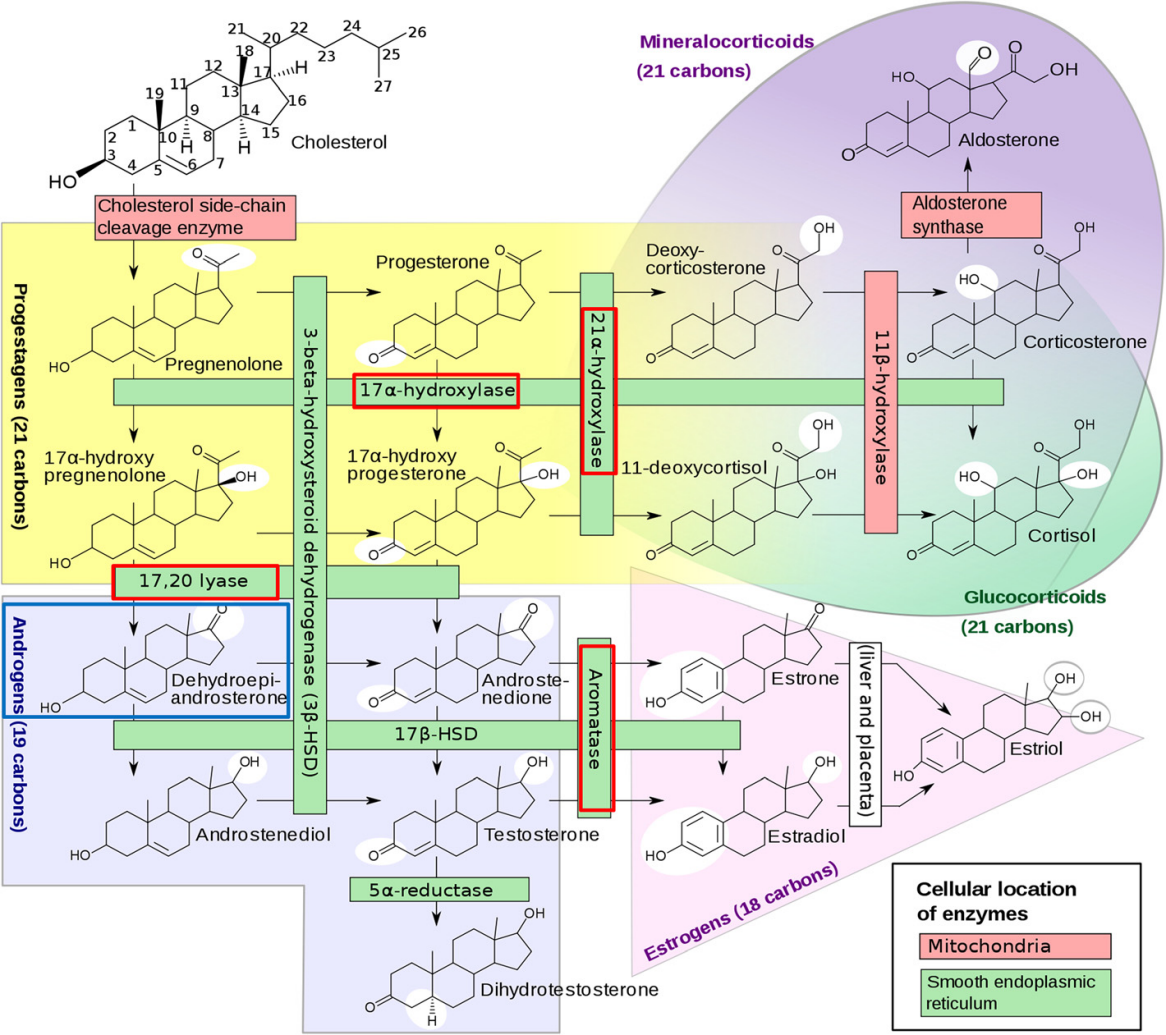
**Steroid synthesis.** Enzymes indicated with a red box have been found in our analysis. CYP17A1 encodes for an enzyme which has both a 17α-hydroxylase and a 17,20 lyase function. CYP21A2 encodes for a steroid 21-hydroxylase and CYP19A1 encodes for an aromatase. Figure derived from the image Steroidogenesis.png in Wikipedia, by David Richfield and Mikael Häggström, licensed under Creative Commons CC BY-SA 3.0 and GFDL.

Interestingly in Figure 3 we also see three cytochrome P450s, i.e. *CYP17A1*, *CYP19A1* and *CYP21A2*, which are key regulator enzymes in the steroid synthesis (Figure 4). The sub-network in Figure 2C shows the three cytocromes P450s and their direct gene neighbors. Analysis of this sub-network showed that many of the genes in the network are mentioned in studies from women suffering of the Polycystic ovary syndrome (PCOS), in which there is an imbalance of a woman's female sex hormones. PCOS is characterized by insulin resistance, possibly because of hyperandrogenism and low levels of SHBG. The latter effect has also been observed in men suffering from the metabolic syndrome [41]. Also a study by Macut *et al*. suggested that alterations of a cross-talk between glucocorticoid signaling and metabolic parameters, is related to PCOS pathophysiology [42].

Additional topological analysis of the sub-network using cytohubba [43] revealed that *IGF1R*, *HSD11B2*, *IGF2* and *SHBG* have a high betweenness centrality, i.e. they have many shortest paths going through them, analogous to major bridges and tunnels on a high map. Studies show that such a bottle necks play important roles in biological networks [44,45].

*CYP19A1* encodes for an aromatase which is responsible for the aromatization of androgens into estrogens, thus influencing the androgen to estrogen balance. Several studies showed that an imbalance between androgen and estrogen balance because of aromatase deficiency resulted in the development of symptoms related to the metabolic syndrome [46-49]. The fact that dexamethasone can regulate aromatase activity [50-52], suggests a role of aromatase in GC induced IR.

*CYP17A1* is a key regulator of androgen synthesis and catalyzes the reactions in which pregnenolone and progesterone are converted into their 17-alpha-hydroxylated products and subsequently into Dehydroepiandrosterone (DHEA). A decline in DHEA and also its sulfated ester (DHEA-S) has been suggested to be causally linked to insulin resistance and obesity [53-56]. The possible inhibitory effects of dexamethasone on *Cyp17a1*[57,58] suggests a role in GC induced IR by this gene.

*CYP21A2* is a cytochrome P450 enzyme coding for the 21-hydroxylase that is involved in the biosynthesis of the steroid hormones aldosterone and cortisol. A defect in this gene leads to Congenital adrenal hyperplasia (CAH) in which there is a disbalance in cortisol and aldosterone secretion. CAH patients are characterized by insulin resistance, lower insulin sensitivity and hyperinsulinemia [43,59-61]. Some studies indicate that the development of IR is because of GC treatment in this patient group [62-64]. Whether these patients develop IR because of CAH and deficiency of 21-hydroxylase, or because of the fact that they are often treated with synthetic GCs need to be elucidated.

### Genes involved in osteoporosis

Another side effect of GC treatment is the development of glucocorticoid induced osteoporosis (GIOP) [65]. GIOP is characterized by reduced bone mineral density (BMD), decreased bone mass and disturbance of the bone matrix, leading to increased susceptibility to fractures. We applied CoPubGene to deduce important genes involved in GIOP by analyzing top scoring genes with OP (in total 131 genes associated with OP were found, see Additional file 2: Table S5; the network of these top scoring genes with relations to dexamethasone and inflammation is shown in Additional file 6: Figure S3. The majority of the genes are involved in bone remodeling and resorption (*TNFRSF11A*, *TNFRSF11B*, *TNFSF11*, *SP7* ,*CTSK*), in bone mineralization (*PTH*, *Klotho*, *VDR*, *Calca*, *BGLAP*) or are part of the wnt signaling pathway that is involved in the regulation of bone formation (*SOST*, *DKK1*, *LRP5*, *LRP6*) [66]. Among these genes are known biomarkers of GIOP such as osteoprotegerin (encoded by *TNFRSF11B*) and the ligand RANK-L (encoded by TNFS11) [67]. Here we also searched for genes with a low score with inflammation. Several of these genes in the set, such as *BGLAP*, *COL1A1* and *SP7* are affected by GCs [68-72], have low associations with inflammation and therefore are interesting biomarker candidates for GIOP.

## Discussion

In the work presented here we used Medline abstracts to study mechanisms and genes involved in glucocorticoid induced insulin resistance. We created CoPubGene, a number of web service operations that can be used to retrieve relevant gene-disease, gene-drug and gene-gene associations out of Medline abstracts, using the CoPub technology.

The clustering of disease terms based on their associations with genes in Medline abstracts showed that CoPubGene is able to generate a list of specific IR genes that can be used for further analysis, and that this method also can be used to generate a variety of other gene disease associations. We used this clustering to evaluate the quality of disease related gene lists, generated using a text mining approach, because to our knowledge there is no real gold standard data set that covers a sufficient range of gene-disease associations that can be used. Databases such as OMIM and the KEGG disease database [73] only cover a sub range of diseases which makes these datasets difficult to use in this type of evaluation.

Next, we studied the IR genes in their functional context, by including genes with which they co-occur in Medline abstracts. In this gene network we focused on genes that are strongly linked to dexamethasone and less strongly to inflammation. These genes are thought to be more exclusively related to GC induced IR and therefore might be interesting markers for this effect.

However, all of them are to a certain extent related to inflammation, either directly or indirectly by their neighbors, which suggests that these genes cannot be used as an exclusive marker for GC induced IR. This might have consequences for the search of dissociating compounds, i.e. compounds which only have the immune suppressive properties and not the unwanted side effects. Instead the search should focus on compounds that show a reduced effect on the expression of these IR genes.

The majority of the IR genes that have a low literature neighbor score for inflammation (< 25) and a high score for dexamethasone (literature neighbor score > 25) code for enzymes and hormones directly involved in important metabolic processes, such as glycolysis, gluconeogenesis, glucose uptake and lipid metabolism. All these processes are tightly regulated by insulin. This suggests that at a first instance, the search for mechanisms of GC induced IR should be focused on these processes.

Additionally, we also identified a sub network of genes involved in sex steroid synthesis that to our knowledge, not have been recognized yet as mediators of GC induced side effects. Key enzymes involved in steroid synthesis, i.e. *CYP17A1*, *CYP21A2* and *CYP19A1* keep the balance between several steroids, and an impairment of this balance could possibly result in metabolic disturbances such as IR. Additional topological analyses could further prioritize this sub-network for follow-up studies to determine the influence of GCs on sex steroid synthesis and the relation to IR. In such a study one could look at the influence of GCs on the balance between the steroids in combination with their influence on insulin stimulated glucose uptake in glucose sensitive tissues such as adipose and muscle tissue.

## Conclusions

Using CoPubGene we are able to construct an informative literature network of IR related genes by only using information from Medline abstracts. Our approach revealed genes, that on a first glance were not considered to be involved in GC induced IR and thus gave new insights that might lead to a better understanding of the mechanisms behind GC induced IR.

## Competing interests

Authors have no conflict of interest.

## Authors’ contributions

WF performed all data analysis, research design and wrote the paper. ET supervised with biological interpretation and helped with writing the paper. SV, TH developed the web service operations. TR, RVS, RF and JDV helped with research design. WA supervised the work and helped with research design, analyzing the data and writing the manuscript. All authors read and approved the final manuscript.

## Financial contribution

Author WF was supported by the Biorange project (BR4.2) “A Systems Bioinformatics Approach For Evaluating And Translating Drug-Target Effects In Disease Related Pathways” of NBIC.

## Supplementary Material

Additional file 1: Table S2 Part of the disease matrix, which has been used for the clustering. Click here for file

Additional file 2: Table S2 384 genes that are linked with insulin resistance in Medline abstracts. **Table S3**. Gene disease profiles. **Table S4A.** Enriched drug terms. Table S4B. Enriched disease terms. **Table S4A.2.** Genes linked with enriched drug terms. **Table S4B.2.** Genes linked with enriched disease terms. **Table S5.** 131 genes that linked with osteoporosis. Click here for file

Additional file 3: Figure S2 Hierarchical cluster of disease terms from the CoPub database with bootstrapping values. Red numbers at the nodes represent Approximately Unbiased (AU) bootstrap values (%). Green numbers at the nodes represent Bootstrap Probability (BP) value (%). Click here for file

Additional file 4 Enriched disease terms found per sub-cluster when searching with the DAVID annotation server. Click here for file

Additional file 5: Figure S1 Distribution of connectivity of IR related gene network. The node connectivity follows a significant power law distribution (p-value < 0.001). Click here for file

Additional file 6: Figure S3 Network of top scoring genes with osteoporosis. Genes in blue have a co-occurrence with dexamethasone in Medline abstracts (R-scaled score). The strength of the link with dexamethasone is given by the color shading, ranging from no link (white) to a strong link (dark blue). The strength of the link with inflammation (R-scaled score) is given by the size of the node of the gene, ranging from no link (normal size of the node) to a strong link with inflammation (large size of the node). Click here for file

## References

[B1] Del Rosso Do JQ Combination topical therapy for the treatment of psoriasis J Drugs Dermatol 2006 5 3 232 234 16573255

[B2] Schwartz M Cohen R Optimizing conventional therapy for inflammatory bowel disease Curr Gastroenterol Rep 2008 10 6 585 590 10.1007/s11894-008-0106-8 19006615

[B3] Hillier SG Diamonds are forever: the cortisone legacy J Endocrinol 2007 195 1 1 6 10.1677/JOE-07-0309 17911391

[B4] De Bosscher K Haegeman G Minireview: latest perspectives on antiinflammatory actions of glucocorticoids Mol Endocrinol 2009 23 3 281 291 19095768 10.1210/me.2008-0283 PMC5428155

[B5] Rhen T Cidlowski JA Antiinflammatory action of glucocorticoids–new mechanisms for old drugs N Engl J Med 2005 353 16 1711 1723 10.1056/NEJMra050541 16236742

[B6] Rockall AG Computed tomography assessment of fat distribution in male and female patients with Cushing's syndrome Eur J Endocrinol 2003 149 6 561 567 10.1530/eje.0.1490561 14640998

[B7] Schacke H Docke WD Asadullah K Mechanisms involved in the side effects of glucocorticoids Pharmacol Ther 2002 96 1 23 43 10.1016/S0163-7258(02)00297-8 12441176

[B8] Schacke H Insight into the molecular mechanisms of glucocorticoid receptor action promotes identification of novel ligands with an improved therapeutic index Exp Dermatol 2006 15 8 565 573 10.1111/j.1600-0625.2006.00453.x 16842594

[B9] Diamond MI Transcription factor interactions: selectors of positive or negative regulation from a single DNA element Science 1990 249 4974 1266 1272 10.1126/science.2119054 2119054

[B10] Schenk S Saberi M Olefsky JM Insulin sensitivity: modulation by nutrients and inflammation J Clin Invest 2008 118 9 2992 3002 10.1172/JCI34260 18769626 PMC2522344

[B11] Kalupahana NS Moustaid-Moussa N Claycombe KJ Immunity as a link between obesity and insulin resistance Mol Aspects Med 2012 33 1 26 34 10.1016/j.mam.2011.10.011 22040698

[B12] Weinstein SP Dexamethasone inhibits insulin-stimulated recruitment of GLUT4 to the cell surface in rat skeletal muscle Metabolism 1998 47 1 3 6 10.1016/S0026-0495(98)90184-6 9440469

[B13] Ideker T Lauffenburger D Building with a scaffold: emerging strategies for high- to low-level cellular modeling Trends Biotechnol 2003 21 6 255 262 10.1016/S0167-7799(03)00115-X 12788545

[B14] Alkema W Rullmann T van Elsas A Target validation in silico: does the virtual patient cure the pharma pipeline? Expert Opin Ther Targets 2006 10 5 635 638 10.1517/14728222.10.5.635 16981820

[B15] Sharan R Ideker T Modeling cellular machinery through biological network comparison Nat Biotechnol 2006 24 4 427 433 10.1038/nbt1196 16601728

[B16] Goh KI The human disease network Proc Natl Acad Sci U S A 2007 104 21 8685 8690 10.1073/pnas.0701361104 17502601 PMC1885563

[B17] Plake C Schroeder M Computational polypharmacology with text mining and ontologies Curr Pharm Biotechnol 2011 12 3 449 457 10.2174/138920111794480624 21133848

[B18] Alako BT CoPub Mapper: mining MEDLINE based on search term co-publication BMC Bioinformatics 2005 6 51 10.1186/1471-2105-6-51 15760478 PMC1274248

[B19] Frijters R CoPub: a literature-based keyword enrichment tool for microarray data analysis Nucleic Acids Res 2008 36 Web Server issue W406 410 18442992 10.1093/nar/gkn215 PMC2447728

[B20] Fleuren WW CoPub update: CoPub 5.0 a text mining system to answer biological questions Nucleic Acids Res 2011 39 Web Server issue W450 454 21622961 10.1093/nar/gkr310 PMC3125746

[B21] Friberg PA Larsson DG Billig H Transcriptional effects of progesterone receptor antagonist in rat granulosa cells Mol Cell Endocrinol 2010 315 1–2 121 130 19818377 10.1016/j.mce.2009.09.030

[B22] Frijters R Prednisolone-induced differential gene expression in mouse liver carrying wild type or a dimerization-defective glucocorticoid receptor BMC Genomics 2010 11 359 10.1186/1471-2164-11-359 20525385 PMC2895630

[B23] Frijters R Literature-based compound profiling: application to toxicogenomics Pharmacogenomics 2007 8 11 1521 1534 10.2217/14622416.8.11.1521 18034617

[B24] Merkl M Microarray analysis of equine endometrium at days 8 and 12 of pregnancy Biol Reprod 2010 83 5 874 886 10.1095/biolreprod.110.085233 20631402

[B25] Mitterhuemer S Escherichia coli infection induces distinct local and systemic transcriptome responses in the mammary gland BMC Genomics 2010 11 138 10.1186/1471-2164-11-138 20184744 PMC2846913

[B26] Shimizu T Actions and interactions of progesterone and estrogen on transcriptome profiles of the bovine endometrium Physiol Genomics 2010 42A 4 290 300 10.1152/physiolgenomics.00107.2010 20876846

[B27] Voice MW Webster AP Burchell A The in vivo regulation of liver and kidney glucose-6-phosphatase by dexamethasone Horm Metab Res 1997 29 3 97 100 10.1055/s-2007-978998 9137977

[B28] Franckhauser S Expression of the phosphoenolpyruvate carboxykinase gene in 3T3-F442A adipose cells: opposite effects of dexamethasone and isoprenaline on transcription Biochem J 1995 305 Pt 1 65 71 7826355 10.1042/bj3050065 PMC1136430

[B29] Huson DH Dendroscope: An interactive viewer for large phylogenetic trees BMC Bioinformatics 2007 8 460 10.1186/1471-2105-8-460 18034891 PMC2216043

[B30] Huang da W Sherman BT Lempicki RA Systematic and integrative analysis of large gene lists using DAVID bioinformatics resources Nat Protoc 2009 4 1 44 57 19131956 10.1038/nprot.2008.211

[B31] Huang da W Sherman BT Lempicki RA Bioinformatics enrichment tools: paths toward the comprehensive functional analysis of large gene lists Nucleic Acids Res 2009 37 1 1 13 10.1093/nar/gkn923 19033363 PMC2615629

[B32] Barabasi AL Scale-free networks: a decade and beyond Science 2009 325 5939 412 413 10.1126/science.1173299 19628854

[B33] Xu H Dual specificity MAPK phosphatase 3 activates PEPCK gene transcription and increases gluconeogenesis in rat hepatoma cells J Biol Chem 2005 280 43 36013 36018 10.1074/jbc.M508027200 16126724

[B34] Park JJ GRB14, GPD1, and GDF8 as potential network collaborators in weight loss-induced improvements in insulin action in human skeletal muscle Physiol Genomics 2006 27 2 114 121 10.1152/physiolgenomics.00045.2006 16849634

[B35] Krempler F Leptin, peroxisome proliferator-activated receptor-gamma, and CCAAT/enhancer binding protein-alpha mRNA expression in adipose tissue of humans and their relation to cardiovascular risk factors Arterioscler Thromb Vasc Biol 2000 20 2 443 449 10.1161/01.ATV.20.2.443 10669642

[B36] Chia YY Amelioration of glucose homeostasis by glycyrrhizic acid through gluconeogenesis rate-limiting enzymes Eur J Pharmacol 2012 677 1–3 197 202 22227336 10.1016/j.ejphar.2011.12.037

[B37] Fernandez-Real JM Serum corticosteroid-binding globulin concentration and insulin resistance syndrome: a population study J Clin Endocrinol Metab 2002 87 10 4686 4690 10.1210/jc.2001-011843 12364459

[B38] Kern PA Adipose tissue tumor necrosis factor and interleukin-6 expression in human obesity and insulin resistance Am J Physiol Endocrinol Metab 2001 280 5 E745 751 11287357 10.1152/ajpendo.2001.280.5.E745

[B39] Weisberg SP CCR2 modulates inflammatory and metabolic effects of high-fat feeding J Clin Invest 2006 116 1 115 124 10.1172/JCI24335 16341265 PMC1307559

[B40] Tamura Y C-C chemokine receptor 2 inhibitor improves diet-induced development of insulin resistance and hepatic steatosis in mice J Atheroscler Thromb 2010 17 3 219 228 10.5551/jat.3368 20179360

[B41] Bhasin S Sex hormone-binding globulin, but not testosterone, is associated prospectively and independently with incident metabolic syndrome in men: the framingham heart study Diabetes Care 2011 34 11 2464 2470 10.2337/dc11-0888 21926281 PMC3198304

[B42] Macut D Age, body mass index, and serum level of DHEA-S can predict glucocorticoid receptor function in women with polycystic ovary syndrome Endocrine 2010 37 1 129 134 10.1007/s12020-009-9277-9 20963561

[B43] Mooij CF Unfavourable trends in cardiovascular and metabolic risk in paediatric and adult patients with congenital adrenal hyperplasia? Clin Endocrinol (Oxf) 2010 73 2 137 146 19719762 10.1111/j.1365-2265.2009.03690.x

[B44] Yu H The importance of bottlenecks in protein networks: correlation with gene essentiality and expression dynamics PLoS Comput Biol 2007 3 4 e59 10.1371/journal.pcbi.0030059 17447836 PMC1853125

[B45] McDermott JE Bottlenecks and hubs in inferred networks are important for virulence in Salmonella typhimurium J Comput Biol 2009 16 2 169 180 10.1089/cmb.2008.04TT 19178137

[B46] Bader MI Comparative assessment of estrogenic responses with relevance to the metabolic syndrome and to menopausal symptoms in wild-type and aromatase-knockout mice J Steroid Biochem Mol Biol 2011 127 (3-5) 428 34 21621614 10.1016/j.jsbmb.2011.05.004

[B47] Jones ME Of mice and men: the evolving phenotype of aromatase deficiency Trends Endocrinol Metab 2006 17 2 55 64 10.1016/j.tem.2006.01.004 16480891

[B48] Maffei L Dysmetabolic syndrome in a man with a novel mutation of the aromatase gene: effects of testosterone, alendronate, and estradiol treatment J Clin Endocrinol Metab 2004 89 1 61 70 10.1210/jc.2003-030313 14715828

[B49] Takeda K Progressive development of insulin resistance phenotype in male mice with complete aromatase (CYP19) deficiency J Endocrinol 2003 176 2 237 246 10.1677/joe.0.1760237 12553872

[B50] Zhao H A novel promoter controls Cyp19a1 gene expression in mouse adipose tissue Reprod Biol Endocrinol 2009 7 37 10.1186/1477-7827-7-37 19393092 PMC2684739

[B51] Simpson ER Estrogen formation in stromal cells of adipose tissue of women: induction by glucocorticosteroids Proc Natl Acad Sci U S A 1981 78 9 5690 5694 10.1073/pnas.78.9.5690 6946508 PMC348829

[B52] Enjuanes A Regulation of CYP19 gene expression in primary human osteoblasts: effects of vitamin D and other treatments Eur J Endocrinol 2003 148 5 519 526 10.1530/eje.0.1480519 12720534

[B53] Koga M Serum dehydroepiandrosterone sulphate levels in patients with non-alcoholic fatty liver disease Intern Med 2011 50 16 1657 1661 10.2169/internalmedicine.50.4682 21841322

[B54] Kurzman ID MacEwen EG Haffa AL Reduction in body weight and cholesterol in spontaneously obese dogs by dehydroepiandrosterone Int J Obes 1990 14 2 95 104 2140342

[B55] Sanchez J Dehydroepiandrosterone prevents age-associated alterations, increasing insulin sensitivity J Nutr Biochem 2008 19 12 809 818 10.1016/j.jnutbio.2007.10.005 18482832

[B56] Schriock ED Divergent correlations of circulating dehydroepiandrosterone sulfate and testosterone with insulin levels and insulin receptor binding J Clin Endocrinol Metab 1988 66 6 1329 1331 10.1210/jcem-66-6-1329 2967305

[B57] Lee TC Miller WL Auchus RJ Medroxyprogesterone acetate and dexamethasone are competitive inhibitors of different human steroidogenic enzymes J Clin Endocrinol Metab 1999 84 6 2104 2110 10.1210/jc.84.6.2104 10372718

[B58] Trzeciak WH Dexamethasone inhibits corticotropin-induced accumulation of CYP11A and CYP17 messenger RNAs in bovine adrenocortical cells Mol Endocrinol 1993 7 2 206 213 10.1210/me.7.2.206 8385739

[B59] Speiser PW Insulin insensitivity in adrenal hyperplasia due to nonclassical steroid 21-hydroxylase deficiency J Clin Endocrinol Metab 1992 75 6 1421 1424 10.1210/jc.75.6.1421 1464643

[B60] Paula FJ Androgen-related effects on peripheral glucose metabolism in women with congenital adrenal hyperplasia Horm Metab Res 1994 26 11 552 556 10.1055/s-2007-1001755 7875653

[B61] Saygili F Oge A Yilmaz C Hyperinsulinemia and insulin insensitivity in women with nonclassical congenital adrenal hyperplasia due to 21-hydroxylase deficiency: the relationship between serum leptin levels and chronic hyperinsulinemia Horm Res 2005 63 6 270 274 10.1159/000086363 15956788

[B62] Kroese JM Pioglitazone improves insulin resistance and decreases blood pressure in adult patients with congenital adrenal hyperplasia Eur J Endocrinol 2009 161 6 887 894 10.1530/EJE-09-0523 19755409

[B63] Charmandari E Children with classic congenital adrenal hyperplasia have elevated serum leptin concentrations and insulin resistance: potential clinical implications J Clin Endocrinol Metab 2002 87 5 2114 2120 10.1210/jc.87.5.2114 11994350

[B64] Bachelot A Long-term outcome of patients with congenital adrenal hyperplasia due to 21-hydroxylase deficiency Horm Res 2007 67 6 268 276 10.1159/000098017 17170529

[B65] den Uyl D Bultink IE Lems WF Glucocorticoid-induced osteoporosis Clin Exp Rheumatol 2011 29 5 Suppl 68 S93 98 22018192

[B66] Issack PS Helfet DL Lane JM Role of Wnt signaling in bone remodeling and repair HSS J 2008 4 1 66 70 10.1007/s11420-007-9072-1 18751865 PMC2504275

[B67] Canalis E Mechanisms of glucocorticoid-induced osteoporosis Curr Opin Rheumatol 2003 15 4 454 457 10.1097/00002281-200307000-00013 12819474

[B68] Kauh E Prednisone affects inflammation, glucose tolerance, and bone turnover within hours of treatment in healthy individuals Eur J Endocrinol 2012 166 3 459 467 10.1530/EJE-11-0751 22180452

[B69] Eastell R Bone formation markers in patients with glucocorticoid-induced osteoporosis treated with teriparatide or alendronate Bone 2010 46 4 929 934 10.1016/j.bone.2009.12.021 20060078

[B70] Zheng HF Spector TD Richards JB Insights into the genetics of osteoporosis from recent genome-wide association studies Expert Rev Mol Med 2011 13 e28 21867596 10.1017/S1462399411001980

[B71] Fu H Osteoblast differentiation in vitro and in vivo promoted by Osterix J Biomed Mater Res A 2007 83 3 770 778 17559111 10.1002/jbm.a.31356

[B72] Advani S Dexamethasone suppresses in vivo levels of bone collagen synthesis in neonatal mice Bone 1997 20 1 41 46 10.1016/S8756-3282(96)00314-6 8988346

[B73] Kanehisa M KEGG for linking genomes to life and the environment Nucleic Acids Res 2008 36 Database issue D480 484 18077471 10.1093/nar/gkm882 PMC2238879

