# Supplementary material for: Identification of new biomarker candidates for glucocorticoid induced insulin resistance using literature mining
Source: BioData Min. 2013 Feb 4;6:2. doi: 10.1186/1756-0381-6-2 (PMC3577498; doi:10.1186/1756-0381-6-2)
Supplement: Additional file 1: Table S2 — Part of the disease matrix, which has been used for the clustering. [file 1756-0381-6-2-S1.doc]

**Table S2** **Part of the disease matrix**, **which has been used for the clustering**

| **A** |  |  |  |  |  |  |  |
| --- | --- | --- | --- | --- | --- | --- | --- |
| **Gene Symbol** | **Gene Name** | **Insulin resistance** | **Diabetes mellitus, type 2** | **Obesity** | **Rheumatoid arthritis** | **Psoriasis** | **Inflammatory bowel disease** |
| *RBP4* | retinol binding protein 4,plasma | 53 | 47 | 46 | 0 | 0 | 0 |
| *GPR83* | G protein coupled receptor 83 | 51 | 48 | 39 | 0 | 0 | 0 |
| *BSCL2* | Bernardinelli Seip congenital lipodystrophy 2 | 51 | 0 | 0 | 0 | 0 | 0 |
| *RETN* | resistin | 51 | 46 | 47 | 0 | 0 | 37 |
| *AGPAT2* | 1 acylglycerol 3 phosphate O acyltransferase 2 | 50 | 0 | 0 | 0 | 0 | 0 |
| *SERPINA12* | serpin peptidase inhibitor,clade A,member 12 | 50 | 0 | 47 | 0 | 0 | 0 |
| *IRS1* | insulin receptor substrate 1 | 50 | 43 | 40 | 0 | 0 | 0 |
| *ADIPOR2* | adiponectin receptor 2 | 50 | 0 | 45 | 0 | 0 | 0 |
| *STEAP4* | STEAP family member 4 | 50 | 0 | 46 | 0 | 0 | 0 |
| *ADIPOQ* | adiponectin,C1Q and collagen domain containing | 50 | 44 | 46 | 0 | 0 | 0 |
| **B** |  |  |  |  |  |  |  |
| **Gene Symbol** | **Gene Name** | **Insulin resistance** | **Diabetes mellitus, type 2** | **Obesity** | **Rheumatoid arthritis** | **Psoriasis** | **Inflammatory bowel disease** |
| *PADI4* | peptidyl arginine deiminase,type IV | 0 | 0 | 0 | 48 | 41 | 0 |
| *FCRL3* | Fc receptor like 3 | 0 | 0 | 0 | 48 | 0 | 0 |
| *NCOA5* | nuclear receptor coactivator 5 | 0 | 0 | 0 | 48 | 0 | 0 |
| *OLIG3* | oligodendrocyte transcription factor 3 | 0 | 0 | 0 | 47 | 0 | 0 |
| *TNFAIP3* | tumor necrosis factor,alpha induced protein 3 | 0 | 0 | 0 | 46 | 45 | 0 |
| *PTPN22* | protein tyrosine phosphatase,non receptor type 22 | 0 | 0 | 0 | 46 | 41 | 39 |
| *HLADRB4* | major histocompatibility complex,class II,DR beta 4 | 0 | 0 | 0 | 45 | 0 | 36 |
| *HLADRB5* | major histocompatibility complex,class II,DR beta 5 | 0 | 0 | 0 | 45 | 0 | 36 |
| *CHI3L1* | chitinase 3 like 1 | 37 | 0 | 0 | 44 | 0 | 43 |
| *HLADRB1* | major histocompatibility complex,class II,DR beta 1 | 0 | 0 | 0 | 44 | 0 | 36 |

A link between a gene and a disease term is given by the R-scaled score. Top scoring genes with insulin resistance are related to other metabolic disease terms (indicated in green) and not to inflammatory disease terms (indicated in red) (**A**). Top scoring genes with rheumatoid arthritis are related to other inflammatory disease terms (shown in green) and not to metabolic disease terms (show in red) (**B**)
