# Supplementary figures and images for: Identification of new biomarker candidates for glucocorticoid induced insulin resistance using literature mining
Source: BioData Min. 2013 Feb 4;6:2. doi: 10.1186/1756-0381-6-2 (PMC3577498; doi:10.1186/1756-0381-6-2)

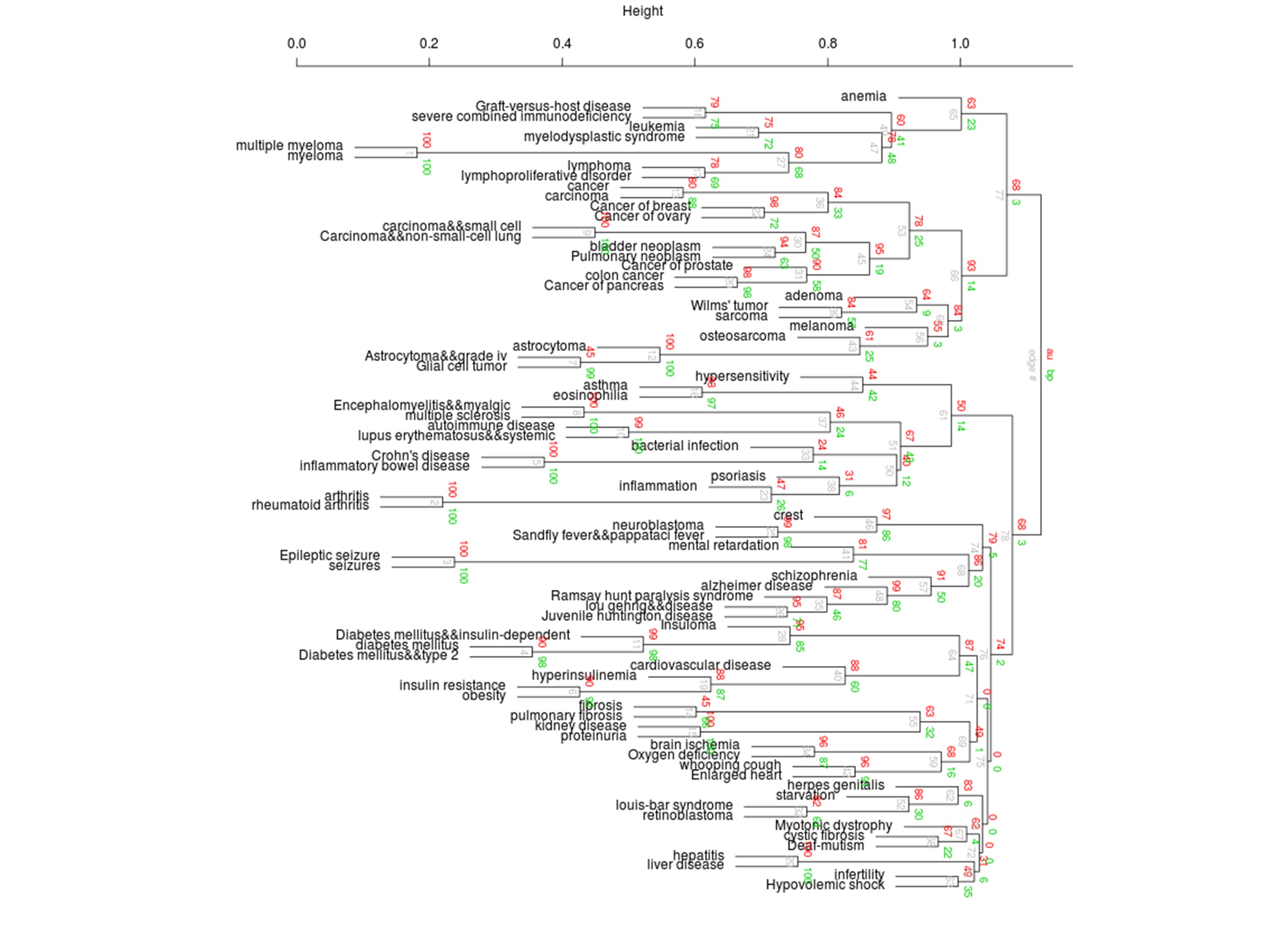

Supplement: Additional file 3: Figure S2 — Hierarchical cluster of disease terms from the CoPub database with bootstrapping values. Red numbers at the nodes represent Approximately Unbiased (AU) bootstrap values (%). Green numbers at the nodes represent Bootstrap Probability (BP) value (%). [file 1756-0381-6-2-S3.png]

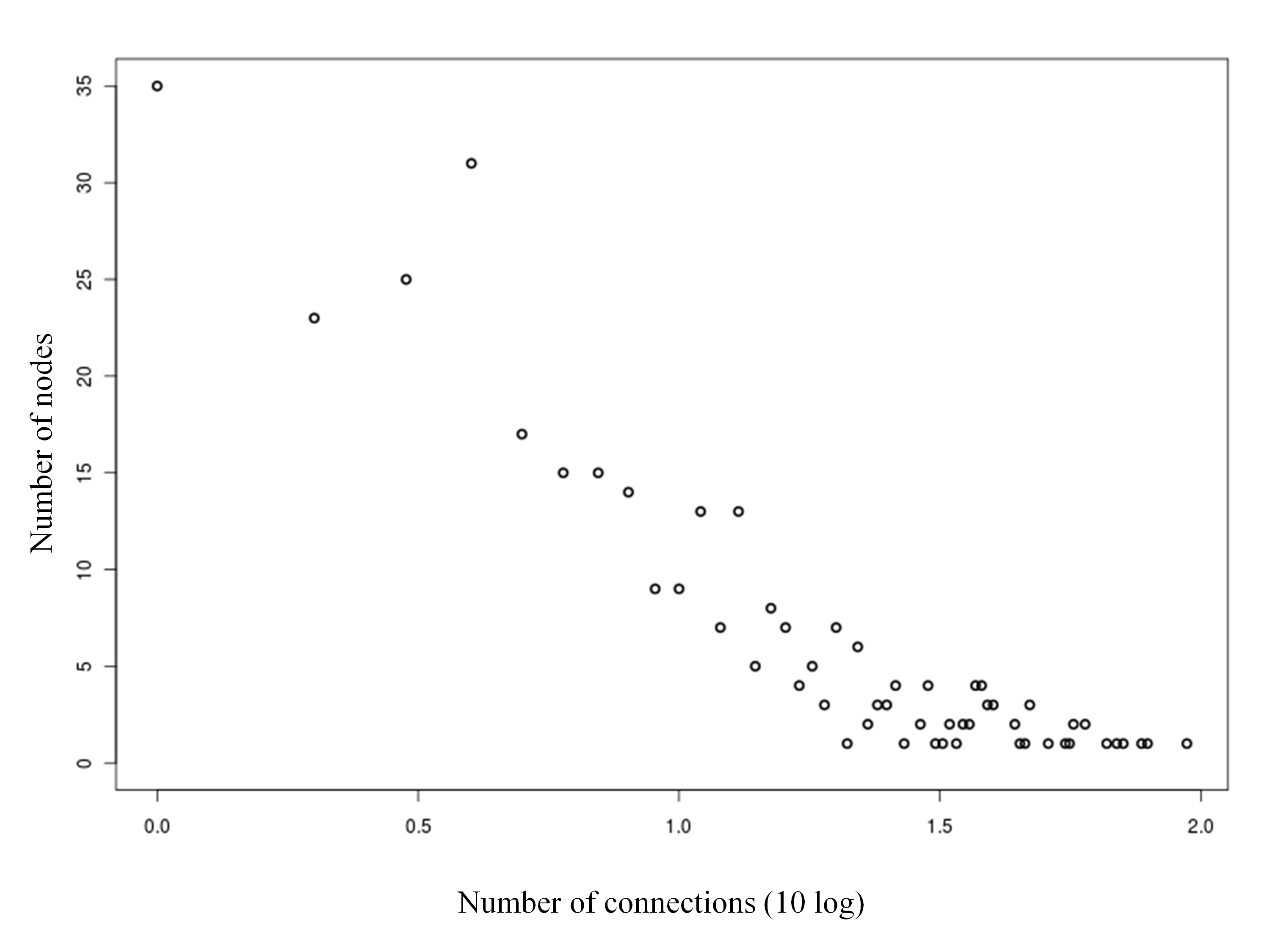

Supplement: Additional file 5: Figure S1 — Distribution of connectivity of IR related gene network. The node connectivity follows a significant power law distribution (p-value < 0.001). [file 1756-0381-6-2-S5.png]

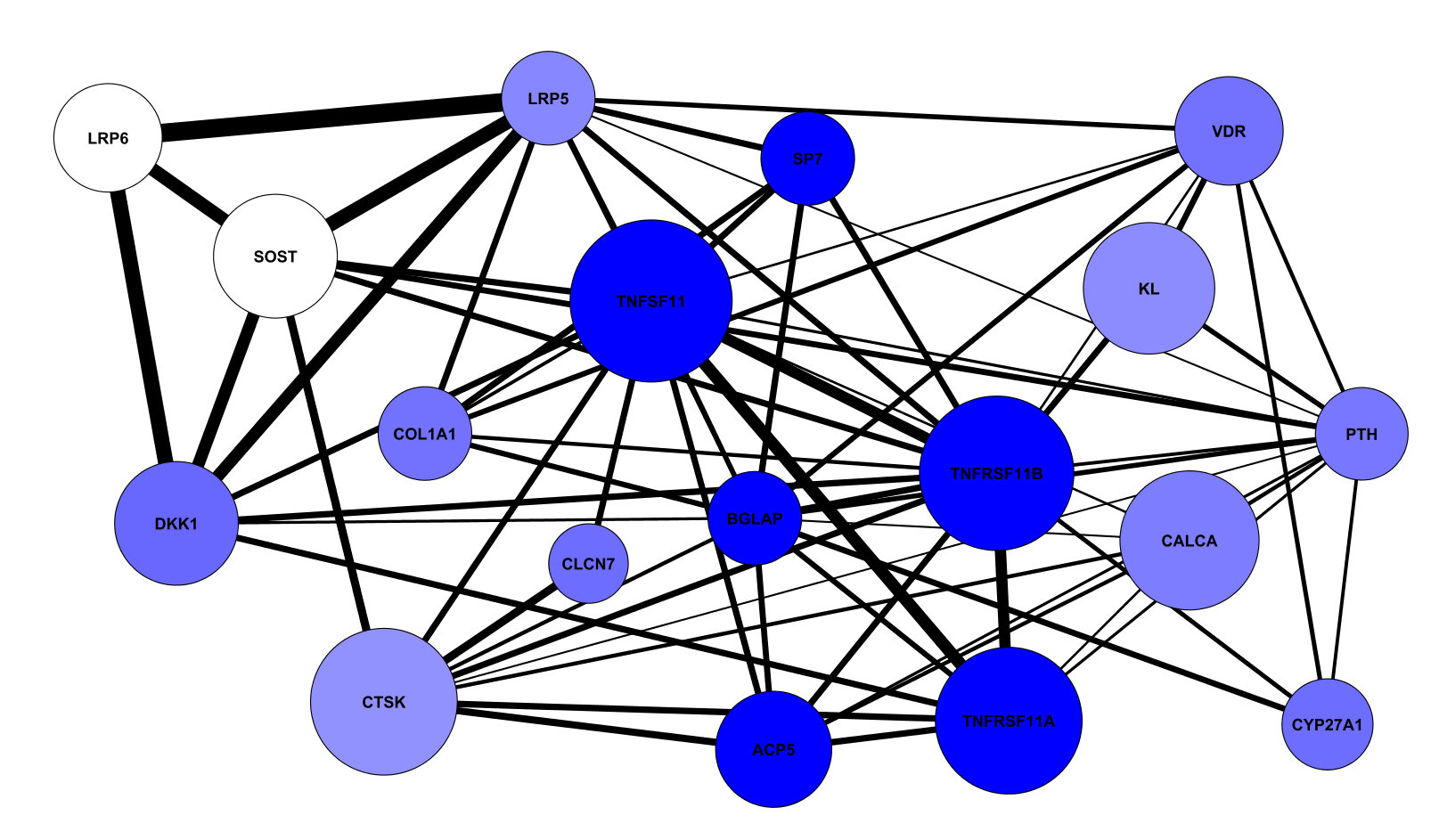

Supplement: Additional file 6: Figure S3 — Network of top scoring genes with osteoporosis. Genes in blue have a co-occurrence with dexamethasone in Medline abstracts (R-scaled score). The strength of the link with dexamethasone is given by the color shading, ranging from no link (white) to a strong link (dark blue). The strength of the link with inflammation (R-scaled score) is given by the size of the node of the gene, ranging from no link (normal size of the node) to a strong link with inflammation (large size of the node). [file 1756-0381-6-2-S6.png]
